# Robot-Guided Ultrasonography in Surgical Interventions

**DOI:** 10.3390/diagnostics13142456

**Published:** 2023-07-24

**Authors:** Răzvan Alexandru Ciocan, Florin Graur, Andra Ciocan, Cosmin Andrei Cismaru, Sebastian Romeo Pintilie, Ioana Berindan-Neagoe, Nadim Al Hajjar, Claudia Diana Gherman

**Affiliations:** 1 Department of Surgery—Practical Abilities, “Iuliu Hațieganu” University of Medicine and Pharmacy Cluj-Napoca, Marinescu Street, No. 23, 400337 Cluj-Napoca, Romania; 2 Department of Surgery, “Iuliu Hațieganu” University of Medicine and Pharmacy Cluj-Napoca, Croitorilor Street, No. 19–21, 400162 Cluj-Napoca, Romania; 3 Research Center for Functional Genomics, Biomedicine and Translational Medicine, “Iuliu Hațieganu” University of Medicine and Pharmacy Cluj-Napoca, Victor Babeș Street, No. 8, 400347 Cluj-Napoca, Romania; 4 “Iuliu Hațieganu” University of Medicine and Pharmacy Cluj-Napoca, Victor Babeș Street, No. 8, 400347 Cluj-Napoca, Romania

**Keywords:** ultrasound, imaging, minimally invasive, robotics

## Abstract

Introduction. The introduction of robotic-guided procedures in surgical techniques has brought an increase in the accuracy and control of resections. Surgery has evolved as a technique since the development of laparoscopy, which has added to the visualisation of the peritoneal cavity from a different perspective. Multi-armed robot associated with real-time intraoperative imaging devices brings important manoeuvrability and dexterity improvements in certain surgical fields. Materials and Methods. The present study is designed to synthesise the development of imaging techniques with a focus on ultrasonography in robotic surgery in the last ten years regarding abdominal surgical interventions. Results. All studies involved abdominal surgery. Out of the seven studies, two were performed in clinical trials. The other five studies were performed on organs or simulators and attempted to develop a hybrid surgical technique using ultrasonography and robotic surgery. Most studies aim to surgically identify both blood vessels and nerve structures through this combined technique (surgery and imaging). Conclusions. Ultrasonography is often used in minimally invasive surgical techniques. This adds to the visualisation of blood vessels, the correct identification of tumour margins, and the location of surgical instruments in the tissue. The development of ultrasound technology from 2D to 3D and 4D has brought improvements in minimally invasive and robotic surgical techniques, and it should be further studied to bring surgery to a higher level.

## 1. Introduction

Minimally invasive surgery techniques require special focus on technical details and improved attention, resulting in a slow learning curve. This is because these surgical techniques are only suitable for selected patients, and the improvement of techniques must take into consideration diverse variables so that the postoperative outcomes of patients are noninferior, but on the contrary, comparable to the classic approach. The introduction of robot-guided procedures increases the accuracy and control of resections. Surgery has evolved as a technique since the development of laparoscopy, which has added different perspectives to the visualisation of the peritoneal cavity. Robotic surgery with a multi-armed robot, such as da Vinci, associated with real-time intraoperative imaging, has improved the manoeuvrability and dexterity of procedures in well-selected cases [1,2].

Compared to mechanical and laparoscopic surgical instruments, robotic devices add to the accuracy of the surgical act, and manoeuvres which have up until now been considered risky can now be safely performed. These devices associated with human thinking can take surgery to a higher level and lead to solving surgical cases that have so far been considered obsolete. One of the most common ways of imaging used in robotic interventions is intraoperative ultrasonography, which can be combined with computed tomography (CT) or magnetic resonance imaging (MRI). The main advantages of ultrasonography are that it is a real-time, non-invasive, inexpensive, and represents an effective method that can visualise anatomical structures in dynamics [3]. Surgeons use ultrasound to visualise abdominal, retroperitoneal, and pelvic structures. In the classical open surgery approach, the surgeon can visualise these structures with the naked eye, palpate them, and use ultrasonography transducers to visualise deep structures of these tissues. Thus, the surgeon can obtain an overview of the patient’s pathology [4].

**Figure 1:**
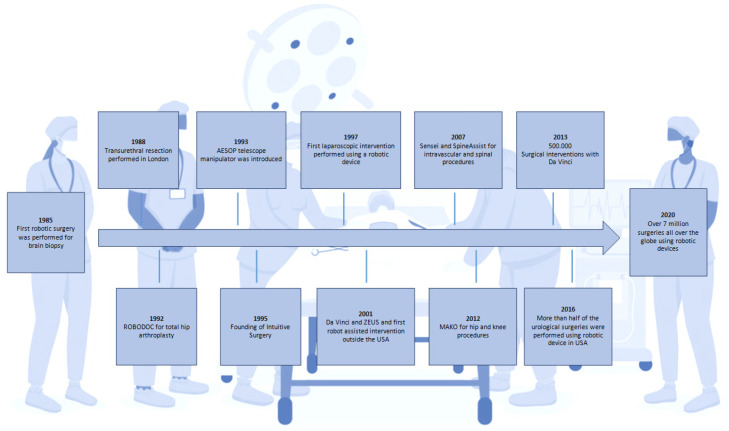
Schematic representation of the major advances in robotic surgery.

In contrast, with the advent of laparoscopy and minimally invasive surgery, the incisions have become considerably smaller than in traditional surgery, and the recovery time, risk of infection, and postoperative discomfort have all lowered significantly. Most of the time, however, tiny incisions render it difficult or impossible to palpate deep structures. The introduction of a transducer for the detailed imaging of anatomical structures seemed inconceivable. To mitigate these shortcomings, hybrid robotic systems associating an imaging component have been developed to restore the surgeon’s comfort and surgical precision [5] (Figure 1). Robotic assistance now provides the surgeon with enormous precision and strength at the end of the robotic arm, while its association with ultrasound imaging provides an in-depth evaluation of tissues. This enables the identification of dissection plans between the densest structures, which is an unprecedented achievement, especially for the removal of malignant tumours where precision oncological surgery can differentiate between tumour-free margins or involved edges. For the treatment of cancer, the implications are even more compelling as cancer has been recognised as a difficult and often untreatable disease even since antiquity. The progresses of surgery can go back to the first description of a tumour which can be found in the Edwin Smith Papyrus, written approximately 5000 years ago, detailing breast cancer. Most, if not all, ancient civilisations referred to various cancers and ways to treat them, from the aforementioned Egyptians to the Romans. Various causes were attributed to this scourge; one of the most influential was Galen’s theory of humours. While Galen himself advised against surgery, other renowned Roman doctors such as Celsus or Pliny the Elder did mention or recommend surgery as a potential therapy. Later on, during the Middle Ages, Arabic doctors developed and described several techniques for the surgical excision of tumours, followed by Western doctors in the years preceding the Renaissance, despite the Catholic Church’s admonition of surgical interventions [6].

Progress in tumour descriptions by pathologists allowed for the development of surgical oncology in the 18th and 19th centuries. During that period, many surgical techniques were first described and introduced, such as radical mastectomy in 1774 [7]. This aggressive approach, often including the removal of not only the entire breast with its skin but also the ipsilateral lymph nodes and part of the pectoralis major muscle, was, however, a breakthrough in the therapy of a previously untreatable disease. In the 19th century, further subspecialisation and the introduction of many procedures was seen: the Billroth I and II techniques, multiple types of colostomies, liver resections, and subtotal- and total thyroidectomy, among others [8].

The development of more advanced medical devices allowed for surgical oncology to extend to previously unavailable organs, such as the lungs. Positive-pressure ventilation finally made thoracic surgery a routine procedure. In 1962, previously advised pneumonectomy was replaced with lobectomy in lung cancer, shown to have the same survival but fewer complications [9]. For breast cancer, progress came later. By the middle of the 20th century, radical mastectomy had even evolved into “extended” radical mastectomy, practised by some surgeons, which included the removal of the internal mammary lymph nodes, supraclavicular lymph nodes, and/or mediastinal lymph nodes using the most radical techniques. In 1990, however, the National Cancer Institute declared conservative surgeries combined with subsequent breast irradiation preferable to previous radical or total mastectomy [10].

**Figure 2:**
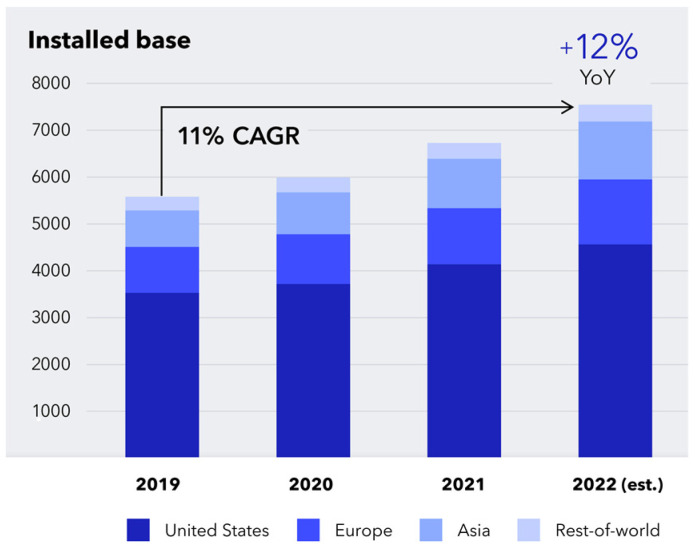
Worldwide distribution of da Vinci robotic surgical systems between 2019 and 2022 (https://isrg.gcs-web.com) (accessed on 12 June 2023).

**Figure 3:**
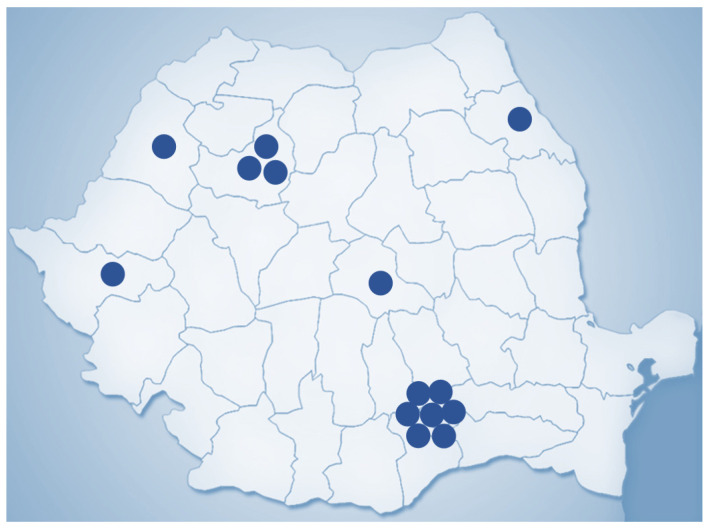
Geographic distribution of da Vinci robotic surgical systems in Romania (2023).

New procedures in recent years include adjuvant treatment such as chemotherapy or radiotherapy, which have paved the way for a multidisciplinary approach to cancer treatment. Relatedly, there has been the progressed implementation of the isolated perfusion of the limbs or even the liver for the targeted administration of toxic chemotherapeutic agents [11]. Most importantly, one of the most modern surgical oncology procedures is robotic assistance to perform minimally invasive surgery. This was first described in 1985, culminating with the FDA’s approval of the da Vinci robot for adult and paediatric surgery in 2000. Initially designed for cardiac surgery, the system now seeded widespread use in many other specialities, including surgical oncology, where it has already led to fewer complications in procedures such as radical prostatectomy or cystectomy [12]. As of 31 March 2023, 7779 robotic surgical systems from the da Vinci family are being placed in hospitals in 70 countries globally, 4668 in the United States and 3111 outside the US, mostly in Europe and Asia (Figure 2). In Romania, there are currently 14 da Vinci robotic surgical systems in both public and private medical sectors (Figure 3).

In thoracic surgery, video-assisted thoracoscopic surgery (VATS) has evolved into robotic-assisted thoracoscopic surgery (RATS), which employs a three-dimensional camera and articulating instruments, which the surgeon can control remotely on a console. This technique can place the camera between the instruments, mimicking the point of view of the surgeon operating with the instruments in his hands [4]. Similar advantages have been described in colorectal cancer surgery, such as three-dimensional, binocular vision, lack of tremor, and better manoeuvrability of tools, which can aid in avoiding sensitive structures such as the hypogastric nerves [13].

On the other hand, several disadvantages have also been pointed out. The lack of tactile feedback may lead to inappropriate sectioning and cutting through the tumour. There is also the increased duration of the surgery and the higher cost which may represent a draw-back [14].

Overall, the use of robotic surgery in surgical oncology is on an ascending curve, with a seven-fold increase in the use of robotic approaches in general surgical oncology in the United States in five years between 2010 and 2014 [15]. The number of robotic systems installed in US hospitals is also on the rise, with the same trend being visible worldwide [16].

In addition to robotic surgery, robot-manipulated ultrasonography helps improve the surgeon’s comprehension of the tumour’s anatomy and location. This approach has been used effectively in various surgical interventions, including liver resections and radical prostatectomy, with positive outcomes in two trials which included 31 and 10 patients, respectively. Ultrasonography handled by robots enables the surgeon’s location of the tumour and surrounding structures, as well as the excision of the malignant lesion with pinpoint accuracy [17,18].

The aim of our study was to provide insights into the development of imaging techniques in robotic surgery in the last ten years with a focus on ultrasonography.

## 2. Materials and Methods

**Figure 4:**
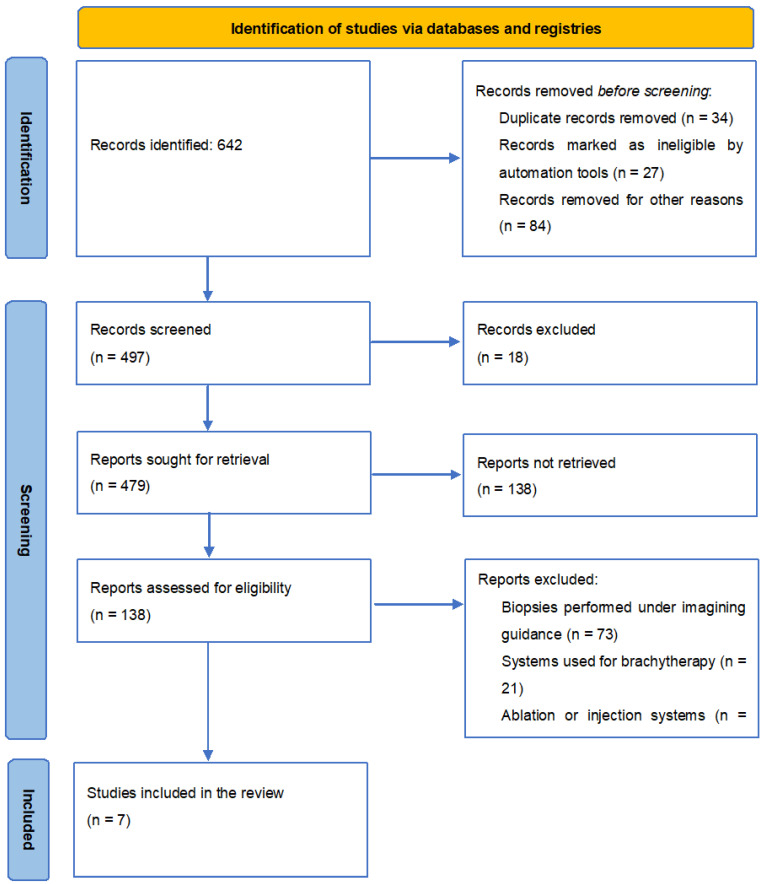
PRISMA 2020 flow diagram for new systematic reviews, which included searches of databases and registers only.

The present study is designed to synthesise the development of imaging techniques in robotic abdominal surgical interventions in the last ten years, with a focus on ultrasonography. To carry out this study, a systematic search of original articles, reviews, clinical trials, and experimental studies was performed on PubMed. The search was performed using the following keywords: “ultrasound”, “imaging”, “minimally invasive”, “robotics”, and “surgery”, using “AND” between keywords as a Boolean Operator. All titles referred to in English and published in a determined period from 2010 to 2019 were checked for eligibility by title and abstract by two researchers to remove double counting. Articles referring to biopsies performed under imaging guidance, systems used for brachytherapy (both autonomous and semi-autonomous systems), and ablation or injection systems were excluded. Also excluded were articles highlighting the use of robotic-guided imaging in urological, gynaecological, and cardiac surgeries or the tracking of surgical instruments, catheters, needles, or other devices used in various surgical techniques which did not fall within the scope of this review. Articles on the use of robotic imaging in abdominal surgery were included in the discussion. The study’s workflow is represented in Figure 4.

## 3. Results

We found 642 results for our keywords. Setting the limit of articles published after 2010 resulted in a total of 479 articles. Also, only full-text articles were selected, resulting in 138 articles. Finally, after applying the inclusion and exclusion criteria, seven articles corresponded to the requirements (Figure 2).

Of the seven studies, two were performed in clinical trials. The other five studies were performed on organs or simulators and attempted to develop a hybrid surgical technique using ultrasonography and robotic surgery.

Patriti et al. (2009) used ultrasound imaging devices in seven cases of colorectal cancer with liver metastases to highlight the vascularisation of the region and the margins of the tumour [19]. Other authors have used imaging devices to identify blood vessels and tumour margins to make a precise resection [19,20] (Table 1).

Most studies aim to surgically identify the blood vessels and nerve structures through this combined technique of surgery and imaging.

Two studies were interested in visualising the tumours and identifying their exact margins to perform the most accurate and correct resection.

One study aimed to inspect organs through this technique and identify structures to highlight the necessity of real-time ultrasonography in surgery.

Another study focused on identifying the type of tissue to assess its status and determine tumour structures at this level.

Araujo et al. (2017) used imaging in laparoscopic robotic interventions to identify vascular lesions. They used an ultrasound transducer into the abdominal cavity placed by an incision in the lower abdominal region in addition to the placement of the usual laparoscopic instruments [26].

Liu et al. (2015) studied the contribution of ultrasonographic imaging for spleen preservation in pancreatoduodenectomies to highlight tumour margins or tumour infiltration at resection time [25]. Giulianotti et al. (2011) studied the importance of laparoscopic surgery in combination with ultrasonography to clamp a series of splenic micro aneurysms. After the intervention, the integrity of blood vessels and blood flow patterns were evaluated using Doppler ultrasound [23].

In terms of the systems used, Schneider et al. (2012) integrated a 2D transducer with the robotic arm and thus performed the liver scan [23]. Other authors used the Da Vinci robot to perform the intervention and inserted an extra 2D linear US probe into the abdomen.

One study used laparoscopic instruments to perform surgery, while the others relied on robotic surgery using the Da Vinci robot. The arms of this robot were adapted, and thus a transducer could be attached to obtain ultrasonographic images.

## 4. Discussion

There are two major groups of imaging applicability in surgery, namely in clinical or experimental studies. These are mainly percutaneous or robotic procedures [26]. Autonomous interventions provide the third direction as an extension of the minimally invasive or robotic ones. In robotic procedures, the surgical technique does not change. What changes is the surgeon’s approach, who does not physically operate near the patient but is in control of a device instead, from which he controls the robot’s arms [27]. Therefore, the operative act acquires high-quality precision, and the human tremor is corrected. So far, these studies have evolved in the direction of helping the surgeon in the operating room. For example, elastography has been used in some cases to guide the surgeon around the tumour mass [28]. In renal surgery, robotic imaging was used to perform partial nephrectomy interventions, thus increasing the visualisation of the structures by the surgeon [29]. These devices cannot yet become completely autonomous because this implies complex procedures that require human interaction and medical reasoning in the moment and quick adaptation to different situations [26]. Another limitation derives from the fact that anatomical imaging is delivered in very small portions, but the development of this aspect could take the idea of autonomy one step further.

Intraoperative navigation combined with ultrasonographic imaging is another goal to achieve because, currently, only hybrid devices exist in laparoscopic surgery. Namely, the laparoscopy camera has a mode of ultrasonography attached. Achieving this combination of real-time images inside the peritoneal cavity and ultrasound images is a complex endeavour [30]. The endoscopic image is 2D and should be adjusted to a 3D space, where the ultrasonographic image should be combined. But the two modes are not performed in the same coordinate system, requiring the application of high-performance image processing techniques in order to identify a structure in both modes and overlap the information from both sources. Also, another current disadvantage would be that laparoscopy uses poor-quality ultrasonographic transducers, and more efficient transducers can be used in open surgery. Another aspect would be that ultrasonography is predominantly 2D, and the surgeon can visualise only a plan at a time and must go step by step to create an overview [31]. Certain programs combine ultrasonographic images in a certain range and make an overview, but this is a slow process and can provide misleading images, unable to process deformed structures. Lately, 3D and 4D ultrasound have been developed and improved together with transducers with a high refresh capacity, which lead to the creation of fast images and much improved quality [32].

Ultrasonography and CT imaging used in minimally invasive interventions are on the rise, necessitating more modifications to become the gold standard in abdominal surgery or any other surgical discipline. Autonomous or robotic systems need automated picture interpretation; this trend will be addressed in the foreseeable future. Currently, more innovative interventions are conducted on experimental organs or simulators. Consecutively, algorithms are being developed to merge pictures so that the surgeon may use these sophisticated approaches to execute difficult procedures with more safety and precision than traditional methods [21]. But transposing these techniques into the clinical environment requires validation, meaning algorithms should work perfectly because, in surgery, any error matters and can have drastic repercussions. This transposition requires time, consistency, and perseverance. The future of autonomous or semi-autonomous systems is now uncertain [33]. It is challenging to create an image that considers the anatomical deformations, position of the surgical instruments, and profound structures. Also, processing these data in real-time is challenging for researchers because it must be fast, exact, and without error.

In addition to identifying the anatomical elements, imaging in surgery should also show the intraoperative position of the surgical instruments. This combination of images is compatible with the operating rooms, does not harm the patient, and has a high chance of being achieved in the future, taking a step forward towards autonomous or semi-autonomous surgical systems [34]. Visualisation techniques have been used for rigid tools such as needles, trocars, and transducers. Robotic surgery would also involve the use of flexible tools [35]. The development of imaging in this direction is essential to locate in real time any type of surgical instrument that the surgeon or robot uses in minimally invasive interventions. Ultrasonography can identify different types of instruments, and by developing this imaging technique, such an approach can potentially achieve semi-autonomous or even autonomous robotic surgeries in the future. Studies have highlighted this concept idea but require significant improvements and changes for them to reach the stage of clinical trials. Three- or four-dimensional ultrasonographic technology combined with tracking imaging techniques, image recording techniques, image fusion techniques, and surgical robot autonomy algorithms is a promising option for the future and should be explored. The final goal of performing autonomous surgeries is difficult to achieve at the moment, but with the development of technology, it may represent a close perspective in the near future [36].

Newer advancements in medical imaging technologies emphasise the acquisition of real-time data. Acquiring photos in real time expedites the process of obtaining a diagnosis and executing a therapy, resulting in vastly improved patient outcomes. This is especially true in surgery, when intraoperative imaging might be critical to the postoperative progression of the patient. This is facilitated by the rise of augmented reality, which fuses computer-generated pictures with actual situations [37].

These opportunities provide new obstacles for the development of new augmented-reality-based approaches. In the future, augmented reality might replace several essential pieces. The surgical applications of augmented reality are still quite restricted. Studies indicate that augmented reality systems have a place in treatment. Augmented reality has the potential to be a revolutionary tool in the field of surgery. It might be created as a human–computer interface to assist surgeons in achieving better outcomes. It is important to have a more precise understanding of the system’s properties and to discover a means to use such a system in routine surgery at a lower cost [38].

## 5. Conclusions

Ultrasonography is often used in minimally invasive surgical techniques. This adds to the visualisation of blood vessels, the correct identification of tumour margins, and the localisation of surgical instruments into the tissues. The surgical technique, which uses imaging during intervention, is compatible with the devices within the operating room, making a real contribution to the procedure. This technique takes robotic minimally invasive interventions to a higher level, achieving an important step in the research towards the implementation of autonomous surgery systems. Most systems described in these studies are still in the development stage but have the potential to reach the level of clinical trials after making necessary adjustments. The development of ultrasound technology from 2D to 3D and 4D has brought improvements for minimally invasive surgical techniques and should be studied. Procedures performed with intraoperative devices, and the combination of imaging with existing operative devices, resulted in hybrid surgical instruments. Their optimisation will lead to the development of the surgical techniques of the future and, probably, to the development of autonomous robotic surgery.

## Figures and Tables

**Table 1:** Purpose and ultrasound probe details of the investigated studies.

Author	Cases (Number)	Purpose	Probe Localisation	Probe Type
Patriti [19]	7	Inspection	Robotic arm	2D US
Giulianotti [21]	9	Blood flow	Robotic arm	Not specific
Schneider [22]	Phantom liver	Vessel localisation	Robotic arm	2D US
Billings [23]	Phantom tissues	Tissue hardness	Robotic arm	2D US
Liu [24]	7	Tumour margins of the pancreas	Robotic arm	2D US
Calin [20]	1	Liver and pancreas vessels	Robotic arm	Not specified
Araujo [25]	Not specified	Liver lesions	Not specified	Not specified

## Data Availability

Not applicable.
